# Supplementary material for: Chaenomeles Fructus (CF), the Fruit of Chaenomeles sinensis Alleviates IL-1β Induced Cartilage Degradation in Rat Articular Chondrocytes
Source: Int J Mol Sci. 2022 Apr 14;23(8):4360. doi: 10.3390/ijms23084360 (PMC9025567; doi:10.3390/ijms23084360)

Supplementary Information

Table S1. Identification and chemical characterization of chemical components authentically or tentatively in the water extracts from *Chaenomeles sinensis* fruit using LC-ESI-QTOF MS/MS

No.	Identified or proposed compound	Formula	mass (Da)	Expected RT (min)	Adduct	Found at mass (Da)	Error (ppm)
6	gallic acid	$C_7H_6O_5$	170.0215	2.71	$[M-H]^-$	169.0144	0.7
7	vanillic acid	$C_8H_8O_4$	168.0423	3.34	$[M-H]^-$	167.0349	-0.2
**1**	**protocatechuic acid**	$C_7H_6O_4$	154.0266	3.86	$[M-H]^-$	153.0195	0.9
8	Neochlorogenic acid	$C_{16}H_{18}O_9$	354.0951	4.02	$[M-H]^-$	353.0875	-0.9
9	Procyanidin dimer 1	$C_{30}H_{26}O_{12}$	578.1424	4.06	$[M-H]^-$	577.1347	-0.7
10	catechin	$C_{15}H_{14}O_6$	290.0790	4.69	$[M-H]^-$	289.0717	-0.2
11	Procyanidin dimer 2	$C_{30}H_{26}O_{12}$	578.1424	4.85	$[M-H]^-$	577.1347	-0.7
12	chlorogenic acid	$C_{16}H_{18}O_9$	354.0951	5.00	$[M-H]^-$	353.0876	-0.7
13	Cryptochlorogenic acid	$C_{16}H_{18}O_9$	354.0951	5.01	$[M-H]^-$	353.0876	-0.7
14	epicatechin	$C_{15}H_{14}O_6$	290.0790	5.67	$[M-H]^-$	289.0718	0.1
15	syringic acid	$C_9H_{10}O_5$	198.0528	6.13	$[M-H]^-$	197.0455	-0.3
16	Procyanidin dimer 3	$C_{30}H_{26}O_{12}$	578.1424	6.48	$[M-H]^-$	577.1350	-0.2
17	Glucopyranosyl-coumaric acid	$C_{18}H_{28}O_{10}$	403.1605	6.62	$[M-H]^-$	403.1606	0.3
**2**	**Quercetin**	$C_{15}H_{10}O_7$	302.0427	9.00	$[M-H]^-$	301.0352	-0.6
**3**	**Luteolin**	$C_{15}H_{10}O_6$	286.0477	9.30	$[M-H]^-$	285.0402	-0.9
18	Betulinic acid	$C_{30}H_{48}O_3$	456.3603	23.34	$[M-H]^-$	455.3531	0.1
**4**	**Oleanolic acid**	$C_{30}H_{48}O_3$	456.3603	25.82	$[M-H]^-$	455.3527	-0.8
**5**	**Ursolic acid**	$C_{30}H_{48}O_3$	456.3603	26.41	$[M-H]^-$	455.3523	-1.8

Figure S1. The extracted ion chromatograms (XICs) of 18 characterized peaks including five components identified with reference standards in the water extract were obtained and identified tentatively using LC-ESI-QTOF MS/MS analysis in negative ion mode

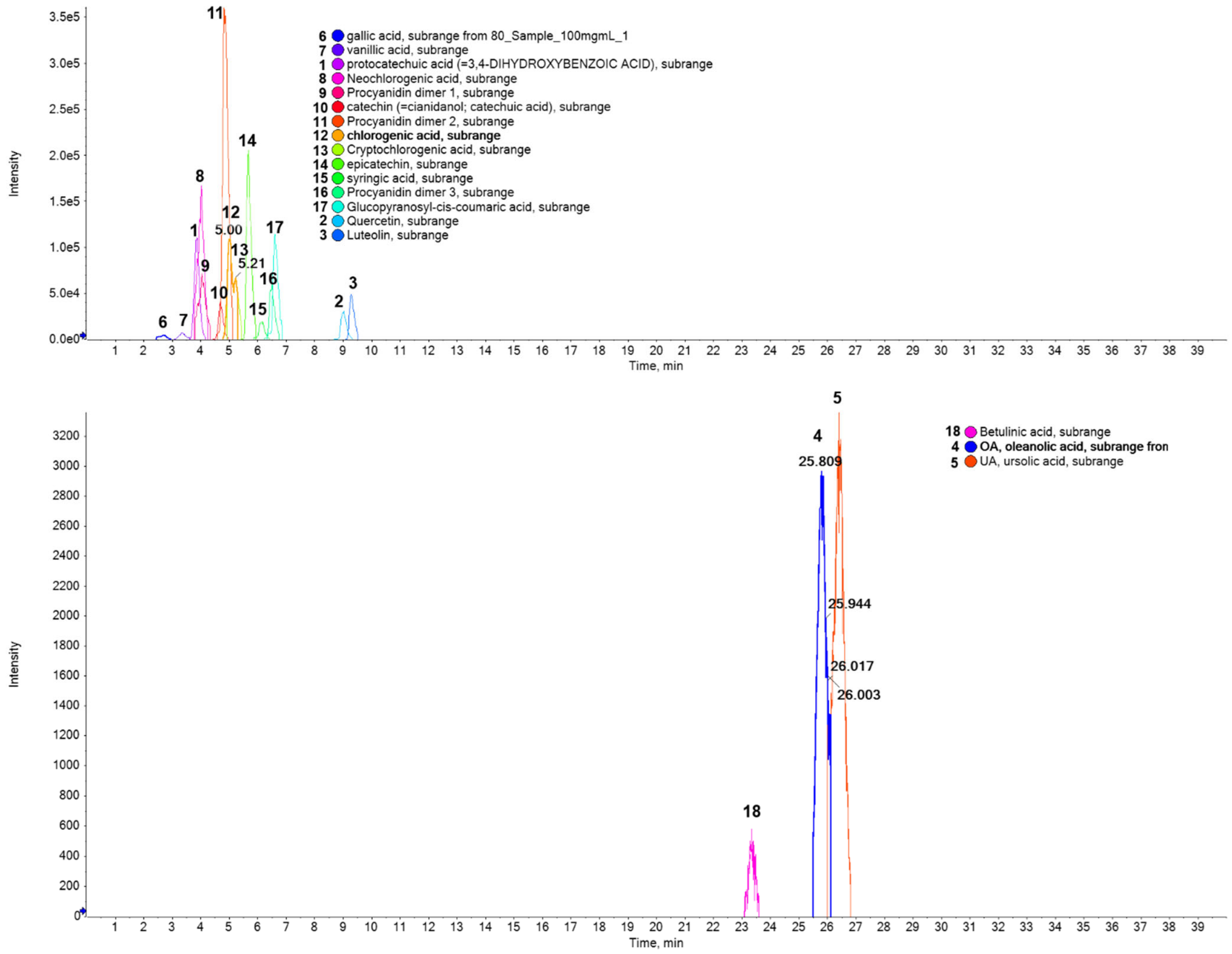

Supplement: Supplementary file 1 [file ijms-23-04360-s001.zip › ijms-1607755-supplementary.pdf]
